# Healthy Lifestyle Behaviors Among Older U.S. Adults With and Without Disabilities, Behavioral Risk Factor Surveillance System, 2003

**Published:** 2006-12-15

**Authors:** Lisa C McGuire, Tara W Strine, Catherine A Okoro, Indu B Ahluwalia, Earl S Ford

**Affiliations:** Division of Adult and Community Health, National Center for Chronic Disease Prevention and Health Promotion, Centers for Disease Control and Prevention; Division of Adult and Community Health, National Center for Chronic Disease Prevention and Health Promotion, Centers for Disease Control and Prevention, Atlanta, Ga; Division of Adult and Community Health, National Center for Chronic Disease Prevention and Health Promotion, Centers for Disease Control and Prevention, Atlanta, Ga; Division of Adult and Community Health, National Center for Chronic Disease Prevention and Health Promotion, Centers for Disease Control and Prevention, Atlanta, Ga; Division of Adult and Community Health, National Center for Chronic Disease Prevention and Health Promotion, Centers for Disease Control and Prevention, Atlanta, Ga

## Abstract

**Introduction:**

Little is known about the relationship between healthy behaviors and the prevalence of chronic diseases in older adults with disabilities. This study examines the prevalence of selected healthy lifestyle behaviors related to chronic diseases among adults aged 65 years and older with and without disabilities.

**Methods:**

Data from the 2003 Behavioral Risk Factor Surveillance System (BRFSS) were used to assess having a healthy weight and six behaviors: current cigarette smoking status, consumption of at least one alcoholic beverage daily, consumption of at least five fruits or vegetables daily, physical activity during the average week, influenza immunization in the past year, and lifetime pneumococcal immunization.

**Results:**

People with a disability were less likely than people without a disability to have a healthy weight (28.5% vs 37.2%) and to engage in the recommended level of weekly physical activity (14.7% vs 26.2%). However, people with a disability were more likely than those without a disability to be nonsmokers (91.8% vs 89.9%), to consume up to one alcoholic beverage daily (95.1% vs 91.5%), to have received their influenza immunization in the past year (72.7% vs 69.0%), and to have received a lifetime pneumococcal immunization (72.1% vs 63.0%). There was no difference between people with and without a disability in the prevalence rates of consuming at least five fruits or vegetables daily.

**Conclusion:**

The prevalence of having a healthy weight and six chronic-disease related behaviors among adults aged 65 years and older varies by disability status and by specific modifiable lifestyle behavior. Screening older adults with and without disabilities and counseling them about health behaviors should be integrated into every interaction between older adults and their health care providers to potentially lower the rates of morbidity and mortality related to chronic diseases in the later years.

## Introduction

Three modifiable lifestyle behaviors — smoking, unhealthy diet, and physical inactivity — have been associated with the development of chronic diseases, specifically heart disease, cancer, stroke, and diabetes (1). Heart disease (31.8%), cancer (21.6%), and stroke (7.9%) were the leading causes of mortality and morbidity in 2002 among U.S. adults aged 65 years and older (2). In addition, participation in healthy behaviors can result in better cognitive functioning (3). Eighty percent of older U.S. adults have at least one chronic condition, 50% have at least two (4), and approximately 95% of older adults' healthcare expenditures are used to treat chronic diseases (1). The economic burdens of chronic disease will continue to grow as the number of older Americans increases.

Chronic disease can result in years of disability and loss of independence (1). Among U.S. adults over the age of 65, 54.5% reported having some type of disability, with 14.2% reporting being unable to perform at least one activity of daily living (ADL) (e.g., bathing, dressing) and 21.6% reporting being unable to perform at least one instrumental activity of daily living (IADL) (e.g., shopping, preparing meals) (5).

Caring for a loved one with a disability has distinct financial implications. In 2000, the average annual costs were estimated to be $13,000 for adult day care, $25,300 for assisted living, and $44,100 for nursing home care (6). In addition, 22.4 million families provided some degree of care for an older adult at an average cost of $109 a day for lost wages and benefits (6).

Despite the abundance of research examining the modifiable health behaviors of older adults in a variety of circumstances (7-20), little is known about the prevalence of healthy lifestyle behaviors among older adults with disabilities. Thus, the goal of our study was to examine the prevalence of six healthy behaviors (including the receipt of influenza and pneumococcal immunizations) and the prevalence of healthy body weight among U.S. adults aged 65 years and older with and without disabilities. A better understanding of healthy behaviors among older adults with disabilities will provide insight that could be used to potentially increase length of expected healthy life and to delay the onset of chronic diseases in this population. 

## Methods

To examine the association between healthy lifestyle behaviors and disability status among adults aged 65 years and older, we analyzed data from the 2003 Behavioral Risk Factor Surveillance System (BRFSS). The BRFSS conducts an ongoing, state-based, random-digit–dialed telephone survey of the noninstitutionalized U.S. population aged 18 years and older and is used to monitor behaviors associated with the leading causes of morbidity and mortality. The BRFSS is in place in all 50 states, the District of Columbia, and the three U.S. territories of Guam, Puerto Rico, and the U.S. Virgin Islands. Information about BRFSS data, survey questions, and modules can be obtained from the BRFSS website (www.cdc.gov/brfss).

Of the 264,684 participants in the 2003 BRFSS survey, 40,414 were aged 65 years and older and were included in our study. Survey participants were considered to have a disability if they answered yes to either of two questions: 1) "Are you limited in any way in any activities because of physical, mental, or emotional problems?"; and 2) "Do you now have any health problem that requires you to use special equipment, such as a cane, a wheelchair, a special bed, or a special telephone?" Participants who responded *don't know/not sure, *refused to answer, or had missing responses for both questions (N = 425) were excluded from the analytical sample.

For this study, we assessed the prevalence of healthy weight (body mass index [BMI] = 18.5–24.9 kg/m^2^) (21) as well as the prevalence of the following six modifiable healthy behaviors: 1) not currently smoking (22); 2) consuming up to one alcoholic beverage per day (23); 3) consuming at least five fruits or vegetables daily (24); 4) engaging in recommended levels of weekly physical activity (25); 5) having received an influenza immunization within the past year (26); and 6) having received a lifetime pneumococcal immunization (27).

BMI was calculated as weight in kilograms divided by the square of height in meters (kg/m^2^) and was used to classify people into four weight categories: underweight (<18.5), healthy weight (18.5–24.9), overweight (25.0–29.9), and obese (≥30.0) (21). Participants were classified by whether or not they met the recommendation for fruit and vegetable intake by their reported consumption of fruit, fruit juice, green salad, potato, carrot, and other vegetable.

Participants were asked how many days in the past 30 days they had at least one drink of any alcoholic beverage and the number of alcoholic beverages consumed, on average, on those days. A drink was considered as one can or bottle of beer, one glass of wine, one can or bottle of wine cooler, one cocktail, or one shot of liquor. Our classification of alcohol consumption is consistent with the National Institute on Alcohol Abuse and Alcoholism (NIAAA) guidelines for clinicians, which define heavy alcohol consumption as exceeding one drink per day for both older men and women (23). We defined current smokers as those who reported currently smoking every day or some days and who had smoked 100 or more cigarettes during their lifetime. 

Meeting recommendations for regular physical activity was determined by asking participants how many days they participated in both moderate- and vigorous-intensity physical activity in a usual week and the duration of activity on those days. Participating in moderate-intensity physical activity (e.g., walking) for at least 30 minutes on 5 or more days per week or vigorous-intensity physical activity (e.g., brisk walking, stair climbing) for at least 20 minutes on 3 or more days per week qualified respondents as meeting physical activity recommendations.

Demographic factors of sex, age, annual income, marital status, education level, race and ethnicity, self-rated health status, health care coverage, and regular care provider were used as covariates and to characterize the population of older adults. Two categories were used for age (65–74 years, ≥75 years), and five were used for income (<$15,000, $15,000–$24,999, $25,000–$34,999, $35,000–$49,999, ≥$50,000) and self-rated health status (excellent, very good, good, fair, poor). Three categories were used for marital status (married or a couple, previously married, never married), education level (<high school diploma, high school diploma, >high school diploma), and race and ethnicity (non-Hispanic white, non-Hispanic black, or other race or ethnicity). Health care coverage was categorized as affirmative if people answered yes to the question, "Do you have any kind of health care coverage, including health insurance, prepaid plans such as HMOs, or government plans such as Medicare?" Participants were considered to have a regular health care provider if they answered yes to the question, "Do you have one person you think of as your personal doctor or health care provider?" If they responded no, they were asked, "Is there more than one or is there no person who you think of?" Participants were then considered to have a regular health care provider if they responded either "yes, only one" or "more than one."

To account for the complex sampling design, SUDAAN software (Research Triangle Institute International, Research Triangle Park, NC) was used in all analyses (28), and only *P* values less than .05 were considered statistically significant. Prevalence rates of specific healthy lifestyle behaviors and of having a healthy weight by disability status were estimated. Logistic regression models were fit independently using participants' disability status as a predictor of each healthy lifestyle behavior and of having a healthy weight. Two models were used, an unadjusted model (Model 1) and a model controlling for age, education level, sex, marital status, race and ethnicity, income, self-rated health status, health care coverage, and regular care provider (Model 2). Odds ratios (OR) and adjusted odds ratios (AOR) for the models for each healthy behavior and for having a healthy weight are provided along with their corresponding 95% confidence intervals (CI).

## Results

Of the 264,684 participants in the 2003 BRFSS survey, 40,414 aged 65 years and older with a reported disability status were included in our analytical sample. Respondents were more likely to be female, aged 65 to 74 years, non-Hispanic white, married, have more than a high school education, and rate their health as very good or good, and approximately half had an annual income of less than $25,000 (Table 1). People with a disability were more likely than those without a disability to be female, be aged 75 years and older, have an annual income of less than $25,000, be previously married, have less than a high school education, rate their health as fair or poor, and have a regular health care provider.

People with a disability were less likely to have a healthy weight or to engage in recommended levels of physical activity in an average week than were those without a disability (Table 2). However, people with a disability were more likely to be nonsmokers, consume no more than one alcoholic drink per day, have received an influenza immunization within the past year, and have had a lifetime pneumococcal vaccination. There was no significant difference found in the unadjusted prevalence by disability status of consuming at least five fruits or vegetables daily. When we stratified by age (65–74 years and ≥75 years), there were no significant differences by disability status in the prevalence of consuming at least five fruits or vegetables daily, being a nonsmoker, and receiving an influenza immunization within the past year.

A series of logistic regression models to assess the association with disability status were independently calculated for the six healthy lifestyle behaviors and having a healthy weight (Table 3). Older adults with a disability were less likely than those without a disability to meet the recommended levels of physical activity in an average week, have a healthy weight, be nonsmokers, and drink one or fewer alcoholic beverages daily. People with a disability had higher odds of receiving an influenza vaccination within the past year and of receiving a lifetime pneumococcal vaccination. There were no differences by disability status in the prevalence of consuming at least five fruits or vegetables daily. When stratified by age group, the disability-related differences in the prevalence of nonsmoking and the receipt of influenza vaccination within the past year were attenuated.

After adjusting the logistic regression models for the covariates of age, education level, sex, marital status, race and ethnicity, income, self-rated health status, health care coverage, and regular care provider, disability-related differences in the prevalence of alcohol consumption and the receipt of influenza vaccination within the past year were attenuated. However, when stratified by age, disability-related differences in the prevalence of alcohol consumption were only attenuated for people who were aged 65 to 74 years but not for those who were aged 75 years and older compared with the unadjusted model.

## Discussion

Adults aged 65 years and older with a disability were less likely to have a healthy weight and to engage in recommended levels of physical activity than people without a disability, but people with a disability were more likely to have received a lifetime pneumococcal immunization and to be nonsmokers than those without a disability. No significant difference between the groups in the likelihood of recommended fruit or vegetable intake, alcohol consumption, and receipt of an influenza vaccination within the previous year was present after adjusting for age, education level, sex, marital status, race and ethnicity, income, self-rated health status, health care coverage, and regular care provider.

A better understanding of behaviors related to chronic disease could lead to improvements in prevention education and care of older adults. In addition, it could decrease the discrepancy between life expectancy and healthy life expectancy, resulting in sustained quality of life for older adults (29). Prior data indicated that at age 65 women have an average life expectancy of 19 years and an average healthy life expectancy of 10 years, while men have an average life expectancy of 14 years and an average healthy life expectancy of 8 years (30). A 12-year longitudinal study demonstrated that people who engaged in more healthy behaviors experienced less disability before death (31). This finding suggests a decrease in morbidity and a longer healthy life expectancy for those who engage in healthy lifestyle behaviors. A better understanding of such behaviors for people with disabilities can be used for the informed planning for future trends in health care as well as the development and implementation of programs that may delay the onset and progression of chronic disease and disability by promoting healthful behaviors and a healthy weight.

Unfortunately, older adults are often not considered an appropriate audience for health promotion activities (32), even though much of the morbidity and mortality related to chronic disease in the United States is attributable to modifiable behaviors (1,10) that could be identified and addressed in primary care settings (10) through screening and counseling. In a recent study, 80% of people aged 65 years and older were found to have at least one chronic health condition (4) and to have visited a physician an average of 6.7 times in the previous year (31). These visits provide opportunities for patients to be screened and counseled about their health behaviors. Even though people aged 65 years and older engage in more healthy lifestyle behaviors than younger people do (8,13,16,17,20), only 46.6% reported being screened for unhealthy lifestyle behaviors during their last routine checkup, a lower percentage than for other age groups (10). However, patient screening and counseling may occur more frequently among certain older adults. For example, people with diabetes are more likely than those without diabetes to be counseled about weight loss (49.8% vs 20.8%), smoking cessation (77.7% vs 67.3%), and increasing their physical activity (67.4% vs 36.0%) (11). One reason that older adults are not screened and counseled more frequently about their healthy behaviors may be because of the high illness burden of older adults, as providers may be focusing on disease management that may or may not be related to their health risk behaviors (10). Nevertheless, more consistent screening and counseling about healthy lifestyle behaviors can aid in the prevention and management of chronic disease and disability in the aging U.S. population.

From 1990 to 2000, the percentage of older adults who exercised and consumed recommended amounts of fruits or vegetables increased, and the percentage of older adults who smoked or consumed more than one alcoholic drink per day decreased (33,34). Unfortunately, among people aged 75 years and older, the prevalence of diagnosed diabetes, high blood pressure, obesity, and being underweight increased between 1990 and 2000 (33). Since specific patterns of healthy lifestyle behaviors tend to occur together (16), the combination of an unhealthy weight and inadequate physical activity might provide the largest opportunity for improvement (12,16). Although some people with disabilities may be physically unable to exercise at the recommended physical activity level, most are able to engage in some types of physical activity (9). Participating in physical activity at lower-than-recommended levels has been shown to slow the progression of functional limitations in older adults with disabilities (35). We found that only 14.7% of people with a disability and 26.2% of those without a disability reported meeting the physical activity recommendations in an average week, and 28.5% of people with a disability and 37.2% of those without a disability had a healthy weight. Therefore, there is substantial room for improvement in these areas. Interestingly, we found no difference in the prevalence of consuming at least five fruits or vegetables daily by disability status, even though the consumption of recommended amounts of fruit or vegetable consumption increased between 1990 and 2000 (33,34).

Adults aged 65 years and older are a unique segment of the United States population, because the majority (99.3%) have insurance coverage through Medicare or Medicaid (36). We found no difference in the prevalence of health insurance coverage between older adults with and without a disability. Previous studies have shown that people who have a regular care provider or a regular place of care were more likely to receive clinical preventive services, such as influenza (15) and pneumococcal immunizations (15,37). In addition, Medicare covers 100% of the cost for influenza and pneumococcal immunizations (38). Consistent with a Medicare Beneficiary Survey (39), we found that 97.6% of older adults reported having a regular care provider and 93.3% had a place of regular care. We found that people with a disability were significantly more likely to have received a lifetime pneumococcal immunization than those without a disability (72.1% vs 63.0%). The higher prevalence rate among those with disabilities may be because of their more frequent interactions with the health care system.

We found that the majority (90.2%) of older adults are nonsmokers, and this is consistent with the trend that the percentage of older adults who smoke decreased between 1990 and 2000 (33,34). However, our results indicate that people with a disability were more likely to be nonsmokers than those without a disability. This finding could be because of selective survival, which can be an issue when studying older adults; there may have been higher morbidity and mortality among those who engaged in fewer healthy lifestyle behaviors, resulting in a surviving population of people who led healthier lifestyles.

Our findings are subject to several limitations. First, the cross-sectional nature of the data does not allow examination of causal relationships between demographic characteristics of older adults and healthy lifestyle behaviors and having a healthy weight. Second, because the BRFSS is a telephone survey, the results may not be generalizable to people aged 65 years and older who do not have telephones; use cell phones exclusively; have difficulties with hearing, speaking, or cognition; have physical limitations that interfere with their ability to answer the telephone; or are institutionalized. Despite this potential limitation, Kinne and Topolski found that adults with disabilities were not any more underrepresented in telephone surveys than in surveys conducted using other methodologies (40). Third, no information was available on the type and degree of disability, which could be related to the healthy lifestyle choices of participants. Despite these limitations, the large sample size of the BRFSS with its proven reliability and validity (41) allowed us to investigate a myriad of healthy lifestyle behaviors in a representative population of older adults.

The prevalence of older adults in the United States who engage in certain modifiable lifestyle behaviors and having a healthy weight vary by disability status. Screening of health behaviors needs to be integrated with every interaction that takes place between older adults and their health care providers. Additionally, counseling older adults about the benefits of healthful behaviors and having a healthy weight may decrease the morbidity and mortality related to chronic diseases and extend healthy life expectancy into the later years.

## Figures and Tables

**Table 1:** Unadjusted Prevalence (SE) of Demographic Characteristics Among U.S. Adults Aged 65 Years and Older, by Disability Status, Behavioral Risk Factor Surveillance System, 2003

Characteristic	No. Respondents	% (SE) (N = 40,414)^a^	Disability Status		*P*

			Has a Disability % (SE) (n = 6667)^a^	Does Not Have a Disability % (SE) (n = 33,662)^a^	
**Sex**					
Male	15,608	44.7 (0.5)	35.2 (1.1)	46.6 (0.5)	<.001
Female	24,806	55.3 (0.5)	64.8 (1.1)	53.4 (0.5)	
**Age, y**					
65–74	23,487	53.7 (0.5)	37.1 (1.1)	56.9 (0.5)	<.001
≥75	16,927	46.3 (0.5)	62.9 (1.1)	43.1 (0.5)	
**Annual income, $**					
<15,000	8675	18.8 (0.4)	31.7 (1.1)	15.4 (0.4)	<.001
15,000–24,999	12,087	28.6 (0.4)	32.8 (1.1)	27.7 (0.5)	
25,000–34,999	7328	18.7 (0.4)	16.0 (0.9)	19.2 (0.4)	
35,000–49,999	5823	15.0 (0.3)	10.3 (0.7)	16.0 (0.4)	
≥50,000	6501	19.6 (0.4)	9.1 (0.6)	21.7 (0.4)	
**Marital status**					
Married/couple	18,742	57.5 (0.5)	41.6 (1.2)	60.7 (0.5)	<.001
Previously married	20,198	39.3 (0.5)	54.3 (1.2)	36.3 (0.5)	
Never married	1474	3.2 (0.2)	4.2 (0.5)	3.0 (0.2)	
**Education level**					
<High school diploma	7372	16.8 (0.3)	23.5 (0.9)	15.5 (0.4)	<.001
High school diploma	14,245	35.0 (0.5)	35.7 (1.1)	34.9 (0.5)	
>High school diploma	18,797	48.1 (0.5)	40.9 (1.1)	49.6 (0.5)	
**Race and ethnicity**					
Non-Hispanic white	34,875	82.4 (0.5)	78.1 (1.2)	83.3 (0.5)	<.001
Non-Hispanic black	2063	7.1 (0.3)	10.6 (0.8)	6.4 (0.3)	
Other	3476	10.5 (0.4)	11.3 (1.0)	10.3 (0.4)	
**Self-rated health status **					
Excellent	4666	11.4 (0.3)	3.7 (0.4)	12.9 (0.3)	<.001
Very good	10,247	26.1 (0.4)	11.6 (0.9)	29.0 (0.5)	
Good	13,513	33.2 (0.4)	25.6 (1.0)	34.7 (0.5)	
Fair	8124	20.1 (0.4)	31.1 (1.1)	17.9 (0.4)	
Poor	3864	9.2 (0.3)	28.0 (1.0)	5.5 (0.2)	
**Heath care coverage**					
Yes	39,594	98.0 (0.1)	97.8 (0.3)	98.0 (0.1)	.51
No	820	2.0 (0.1)	2.2 (0.3)	2.0 (0.1)	
**Regular care provider**					
Yes	37,660	93.3 (0.3)	95.9 (0.6)	92.8 (0.3)	<.001
No	2754	6.7 (0.3)	4.1 (0.6)	7.2 (0.3)	

a Weighted estimate.

**Table 2:** Weighted Prevalence (SE) of Participation in Six Healthy Lifestyle Behaviors and Having a Healthy Weight Among U.S. Adults (N = 40,414) Aged 65 Years and Older, by Disability Status and Age Group, Behavioral Risk Factor Surveillance System, 2003

Behavior/Age, y	No. Respondents	% (SE) (N = 40,414)^a^	Disability Status		*P*

			Has a Disability % (SE) (n = 6667)^a^	Does Not Have a Disability % (SE) (n = 33,662)^a^	
Consumes at least five fruits or vegetables daily	12,020	30.4 (0.4)	29.6 (1.1)	30.5 (0.5)	.88
65–74	6489	28.5 (0.5)	27.5 (1.6)	28.6 (0.6)	.33
≥75	5531	32.6 (0.7)	30.9 (1.4)	33.1 (0.8)	
Engages in recommended levels of physical activity in average week^b^	9847	24.3 (0.3)	14.7 (0.8)	26.2 (0.5)	<.001
65–74	6214	26.6 (0.5)	16.1 (1.3)	27.9 (0.6)	<.001
≥75	3633	21.7 (0.7)	13.8 (1.1)	24.0 (0.8)	
Nonsmoker	36,093	90.2 (0.3)	91.8 (0.6)	89.9 (0.3)	.005
65–74	20,273	87.6 (0.4)	88.5 (1.0)	87.4 (0.4)	.24
≥75	15,820	93.3 (0.4)	93.8 (0.8)	93.1 (0.5)	
Consumes one alcoholic beverage or less daily	37,559	92.1 (0.3)	95.1 (0.5)	91.5 (0.3)	<.001
65–74	21,584	90.8 (0.4)	93.1 (1.1)	90.4 (0.4)	<.001
≥75	15,975	93.6 (0.4)	96.3 (0.5)	92.8 (0.5)	
Receipt of an influenza vaccination within the previous year	28,249	69.6 (0.4)	72.7 (1.0)	69.0 (0.5)	<.001
65–74	15,435	64.9 (0.6)	67.7 (1.7)	64.6 (0.6)	.13
≥75	12,814	75.0 (0.7)	75.6 (1.2)	74.8 (0.8)	
Receipt of a pneumococcal vaccination	26,059	64.5 (0.5)	72.1 (1.1)	63.0 (0.5)	<.001
65–74	13,698	57.8 (0.6)	67.2 (1.7)	56.6 (0.6)	<.001
≥75	12,361	72.1 (0.7)	75.0 (1.3)	71.3 (0.8)	
Healthy weight^c^	14,632	35.8 (0.4)	28.5 (1.0)	37.2 (0.5)	<.001
65–74	7489	30.8 (0.6)	21.1 (1.5)	32.1 (0.6)	<001
≥75	7143	41.5 (0.7)	33.4 (1.4)	43.9 (0.9)	

a Weighted estimate.

b At least 30 minutes of moderate-intensity physical activity on 5 or more days per week or at least 20 minutes of vigorous-intensity physical activity on 3 or more days per week.

c Healthy weight indicates a body mass index (BMI) between 18.5 and 24.9 kg/m^2^.

**Table 3:** Odds Ratios and Adjusted Odds Ratios for Participation in Healthy Lifestyle Behaviors and Having a Healthy Weight Among U.S. Adults (N = 40,414) Aged 65 Years and Older, by Disability Status and Age Group, Behavioral Risk Factor Surveillance System, 2003

Behavior/Age, y	OR (95% CI)			AOR (95% CI)^a^		

	Has a Disability(n = 6667)	Does Not Have a Disability (n = 33,662)	*P*	Has a Disability (n = 6667)	Does Not Have a Disability (n = 33,662)	*P*
Consumes at least five fruits or vegetables daily	0.96 (0.86–1.07)	1.00	.43	.01 (0.89–1.15)	1.00	.86
65-74	0.94 (0.80–1.12)	1.00	.51	1.06 (0.88–1.27)	1.00	.57
≥75	0.90 (0.78–1.05)	1.00	.18	1.00 (0.85–1.18)	1.00	.98
Engages in recommended levels of physical activity in average week^b^	0.48 (0.42–0.56)	1.00	<.001	0.68 (0.59–0.80)	1.00	<.001
65–74	0.50 (0.41–0.60)	1.00	<.001	0.70 (0.56–0.86)	1.00	.001
≥75	0.51 (0.41–0.62)	1.00	<.001	0.67 (0.55–0.83)	1.00	<.001
Nonsmoker	0.79 (0.67–0.94)	1.00	.01	0.70 (0.58–0.85)	1.00	<.001
65–74	0.91 (0.74–1.10)	1.00	.33	0.67 (0.53–0.84)	1.00	<.001
≥75	0.90 (0.66–1.21)	1.00	.48	0.74 (0.55–0.98)	1.00	.04
Consumes one alcoholic beverage or less daily	0.55 (0.43–0.70)	1.00	<.001	0.87 (0.66–1.15)	1.00	.33
65–74	0.70 (0.49–1.00)	1.00	.047	1.09 (0.73–1.63)	1.00	.66
≥75	0.50 (0.36–0.68)	1.00	<.001	0.69 (0.48–1.00)	1.00	.05
Receipt of an influenza vaccination within the previous year	1.20 (1.07–1.33)	1.00	.001	1.12 (0.99–1.28)	1.00	.08
65–74	1.15 (0.98–1.36)	1.00	.09	1.09 (0.90–1.32)	1.00	.35
≥75	1.04 (0.89–1.21)	1.00	.60	1.18 (0.98–1.41)	1.00	.07
Receipt of a pneumococcal vaccination	1.52 (1.36–1.70)	1.00	<.001	1.22 (1.08–1.39)	1.00	.002
65–74	1.57 (1.34–1.84)	1.00	<.001	1.26 (1.05–1.51)	1.00	.01
≥75	1.21 (1.03–1.41)	1.00	.02	1.23 (1.03–1.45)	1.00	.02
Healthy weight^c^	0.68 (0.61–0.76)	1.00	<.001	0.60 (0.53–0.68)	1.00	<.001
65–74	0.55 (0.43–0.71)	1.00	<.001	0.64 (0.53–0.78)	1.00	<.001
≥75	0.64 (0.56–0.74)	1.00	<.001	0.58 (0.50–0.68)	1.00	<.001

OR indicates odds ratio; CI, confidence interval; AOR, adjusted odds ratio.

a Adjusted for age, education level, sex, marital status, race and ethnicity, annual income, self-rated health status, health care coverage, and regular care provider.

b At least 30 minutes of moderate-intensity physical activity on 5 or more days per week or at least 20 minutes of vigorous-intensity physical activity on 3 or more days per week.

c Healthy weight indicates a body mass index (BMI) between 18.5 and 24.9 kg/m^2^.
